# Mechanical Enhancement of Core-Shell Microlattices through High-Entropy Alloy Coating

**DOI:** 10.1038/s41598-018-23857-7

**Published:** 2018-04-03

**Authors:** James Utama Surjadi, Libo Gao, Ke Cao, Rong Fan, Yang Lu

**Affiliations:** 1 Department of Mechanical and Biomedical Engineering, City University of Hong Kong, Kowloon, Hong Kong; 2 Centre for Advanced Structural Materials (CASM), Shenzhen Research Institute, City University of Hong Kong, Shenzhen, 518057 China

## Abstract

Mechanical metamaterials such as microlattices are an emerging kind of new materials that utilize the combination of structural enhancement effect by geometrical modification and the intrinsic properties of its material constituents. Prior studies have reported the mechanical properties of ceramic or metal-coated composite lattices. However, the scalable synthesis and characterization of high-entropy alloy (HEA) as thin film coating for such cellular materials have not been studied previously. In this work, stereolithography was combined with Radio Frequency (RF) magnetron sputtering to conformally deposit a thin layer (~800 nm) of CrMnFeCoNi HEA film onto a polymer template to produce HEA-coated three-dimensional (3D) core-shell microlattice structures for the first time. The presented polymer/HEA hybrid microlattice exhibits high specific compressive strength (~0.018 MPa kg^−1^ m^3^) at a density well below 1000 kg m^−3^, significantly enhanced stiffness (>5 times), and superior elastic recoverability compared to its polymer counterpart due to its composite nature. The findings imply that this highly scalable and effective route to synthesizing HEA-coated microlattices have the potential to produce novel metamaterials with desirable properties to cater specialized engineering applications.

## Introduction

Material development have solely relied on the alteration of its composition for centuries, using techniques such as alloying metals to produce a desired property. Although its effective in most cases, this approach is time consuming. In the recent years, periodically architected micro/nanolattices which harness the effect of structural geometry are of extensive interest owing to its immense potential in breaching the coupling between strength and density, allowing materials with unprecedented properties to be synthesized. Such properties include high-strength, ultra-low density, negative Poisson’s ratio, high resilience, and energy absorption^1–12^. These cellular materials are three-dimensional assemblies consisting of interconnected beams with micro/nano-sized dimensions designed to induce unique properties onto a material by combining architecture with the inherent properties of its material constituents. They have the potential to be applied across a wide range of science and engineering disciplines, such as building or reinforcement materials in construction, functional materials for electronic devices or energy storage, and bio-scaffolds for cell culturing in biomedicine. Recent successes in the synthesis of architected mechanical metamaterials typically involves a two-stage process: (i) synthesis of a 3D architected polymer scaffold, and (ii) subsequent deposition of a thin film coating, usually consisting of either ceramic, metals or alloys. The mechanical enhancement due to coating could be attributed to the intrinsic properties of the coating material or the ability to exploit the “size effect” typically exhibited in nano-scale materials. Examples of the coating materials used includes Ni-P^6,9^, Ni-B^13^, Al_2_O_3_^7^, Cu^2^, Au^14^, Si-N^15^, and metallic glass^16^. Nevertheless, it is still an ongoing challenge to discover new materials that can be easily coupled with architected micro/nanolattices to develop novel materials with remarkable characteristics, such as high strength-to-weight ratio. The limited capabilities of current advanced manufacturing technologies (e.g. 3D-printing) also poses a challenge for the fabrication of large-scale micro/nanolattices with many unit cells despite its ability to produce topologically complex structures, unattainable using traditional casting or molding methods. However, technological advances in the field of additive manufacturing made during the past few years have demonstrated its potential to be implemented industrially, especially light-based additive manufacturing^9,17,18^.

High entropy alloys (HEAs), which are barely thirteen years old, have garnered increasing attention recently as a new type of alloy owing to their distinct compositions, microstructures, and tunable properties^19–22^. HEAs are complex alloys which typically consist of five or more core elements, whereas conventional alloys are based on a principal element and different elements are added smaller concentrations to the base element to enhance its properties. This alloy design concept has induced the emergence of novel advanced materials with special properties unachievable by traditional alloys due to the cocktail effect, such as outstanding high/low-temperature strength, high hardness and stiffness, excellent wear resistance, and good corrosion or oxidation resistance^23–26^. The uniqueness of HEAs which enables them to exhibit such properties is primarily attributed to its ability to stabilize as a single-phase crystalline structure despite containing multiple crystal structures through entropy maximization. Furthermore, the fabrication of HEAs does not normally require the use of highly specialized equipment or techniques, thus they can easily be scaled for real-life applications. An example of such HEA is the CrMnFeCoNi HEA, which is a single-phase five element HEA with equiatomic composition of its principal elements. Its mechanical properties have recently been studied by several researchers and an interesting feature characteristic of the CrMnFeCoNi HEA was found, in which it exhibits increased mechanical strength and ductility at low temperatures, especially at cryogenic temperatures. This is ascribed to the transition in its deformation behavior, from planar-slip dislocation at room temperature, to mechanical nanotwinning at low temperatures^27–29^. The CoCrFeNiAl_x_ HEA family allows the alloy properties to be significantly modified by changing the concentration of Al. The rapid advancement of film deposition techniques over the years has made it possible to fabricate nanoscale HEA film onto a substrate at the macro-scale. Among the possible methods to fabricate HEA films, sputtering is known to be the most widely used owing to its quick and efficient synthesis procedure. The successful fabrication of the CoCrFeCuNi, AlCoCrCuFeNi, CoCrFeNiAl_0.3_, and AlCoCrCu_0.5_NiFe HEA films have previously been reported through sputtering^30–34^. Despite the tremendous potential of HEAs as candidates for reinforcement coating materials, the scalable combination of a thin HEA film with 3D architected microlattice structures has not yet been reported.

In this study, we report the synthesis, characterization, and mechanical properties of a core-shell microlattice structure combining HEA (CrMnFeCoNi) film coating with 3D polymeric core for the first time, which contain structural features spanning across seven orders of magnitude, from nanometers to centimeters via employing RF magnetron sputtering and stereolithography. The fabricated hybrid microlattice exhibited high specific strength, reaching up to 0.018 MPa kg^−1^ m^3^, which is higher than or comparable to other alloy or ceramic-coated microlattice, previously known to have the highest strength and stiffness.

## Methods

### Synthesis of HEA-Coated Core-Shell Microlattices

The stretching-dominated FCC lattices were synthesized by employing an additive manufacturing technique based on stereolithography (Nova3D Printer). Initially, the FCC lattice structure was designed by using a commercial Computer-Aided Design (CAD) software, SolidWorks. Subsequently, the Standard Tessellation Language (STL) file of the CAD model was processed by using a slicer and host control software, Creation Workshop to generate the sequence of 2D layers prior to 3D printing. The polymer lattices were then synthesized layer-by-layer using an acrylate-based UV photosensitive resin (L101, Nova3D), mainly composed of non-toxic acrylic polyester and curable using a 405 nm light. The slicing distance was set at 30 µm, with a curing time of 8000 ms for each 2D layer. The 3D geometry of the polymer lattices was chosen to be 3 unit cells wide by 3 unit cells long by 3 unit cells tall. The overall size of the fabricated lattices was 7.50 × 7. 50 × 7.50 mm, with strut diameter being 450 µm and slicing distance of 45 µm. Considering the experimental practicalities, FCC structure was designed for this work to optimize the openness of the unit cells for sputter deposition, while maintaining a reasonably weight-efficient geometry for structural applications owing to its stretching-dominated nature^35,36^. The relative sizes of strut diameter and overall geometry of the lattice structure was chosen as such to ensure that the individual struts have reasonable aspect ratio, since overly long struts can become unstable when printed.

Following stereolithography, the polymer lattices were ultrasonically cleaned by repeated immersion in ethanol solution, succeeded by distilled water for 1 min each until a cloudy emulsion was no longer formed. The samples were then dried in a critical point dryer. RF magnetron sputtering was used to conformally coat a thin HEA film onto the polymer scaffold at 1 × 10^−5^ Pa. The argon flow for ignition was set to be 22 standard cubic centimeters per minute (sccm), while the total argon flow rate was fixed at 12 sccm for 60 min. The RF power used was 500 W, and the substrate was rotated at a rate of 2 rpm min^−1^ to assure uniform film deposition^37^.

### Surface Profile and Mechanical Properties of Reference HEA Film

The film uniformity of the reference HEA film deposited onto the silicon wafer was analyzed macroscopically and microscopically via eye observation and white light interferometry (WLI) using Wyko NT9300 Surface Profiler, respectively. The surface roughness of the film was calculated by scanning random areas measuring 670 × 896 µm each on the coated silicon wafer and averaging the mean roughness (Ra) obtained. The representative 2D surface profile of the HEA film was obtained by measuring the average 2D profiles across the X and Y axis for each scanned area.

The Young’s modulus and Hardness of the reference HEA film deposited was measured through nanoindentation experiments using Hysitron TI950 Tribo Indenter. A Berkovich indenter was employed to measure the mechanical properties of the film at 5 different places and depths near the center of the coated silicon wafer to prevent edge effects. The depths tested were 100 nm, 150 nm, 200 nm, 250 nm, and 300 nm. Multiple depths were tested to observe any potential size effect on the mechanical properties of the film.

### Microstructural and Compositional Characterizations

To conduct a detailed examination of the microstructure and composition of the HEA film, a thin layer of HEA was deposited onto a silicon wafer using RF sputtering. The operating parameters used were the same as when the HEA film was coated onto the polymer scaffold. The microstructure and compositional characterization of the microlattices and reference HEA film were observed by using Field Emission Scanning Electron Microscopy, (FESEM, FEI^TM^ Quanta FEG450) equipped with Energy Dispersive X-Ray Spectroscopy (Oxford^TM^ EDX), and Transmission Electron Microscopy (TEM, JEOL^TM^ JEM 2100 F) for both imaging and selected area electron diffraction (SAED), where the TEM foils were prepared via ion-milling at a temperature of −50 °C to prevent crystallization.

### Finite Element Analysis (FEA) of the Polymer and HEA-Coated Microlattices

FEA was carried out using Abaqus/CAE 2017 software to identify the region of highest stress concentration and stress distribution on the designed FCC microlattice structure, allowing us to predict the areas in which fracture is most likely to occur. The FE simulations were performed by using a static structural analysis in the elastic region of the materials. Initially, the geometrical model was imported from SolidWorks by exporting it as a Standard for the Exchange of Product Data (STEP) file. The dimensions of the structure were identical to the STL file used for 3D-printing. The elastic components of the photopolymer and CrMnFeCoNi HEA were then input into the material database as isotropic materials, which were obtained from the material data sheet and experimental data, respectively. For the composite microlattice, the HEA coating was simulated as a skin with the shell thickness specified as 800 nm, which is the same as the real samples used for experimental testing. Subsequently, the structure was meshed by using the bottom-up approach consisting of 10-node quadratic tetrahedral elements. The quality of the meshed structure was checked by using a refinement study through comparison with other mesh sizes.

To simulate the uniaxial compression on the meshed FCC lattice structures, boundary conditions were applied at both the top and bottom surfaces. The bottom face, which is the surface in contact with the stationary cylinder, was fixed in all the principal directions. On the other hand, the top face, which is the surface that will be in contact with the moving cylindrical rod used to compress the structure, was free to move in the direction of the applied load, namely the z-direction, but is restricted to move in the x- and y-directions to neglect the effect of sliding, which can be considered to be negligible in this case. The compression was simulated by linearly displacing the top surface along the negative z-direction over 50 sub steps until a total strain of 0.03 was reached (close to the elastic limit of the photopolymer).

### *In Situ* Mechanical Characterization of HEA-Coated Microlattices

The experimental setup for the *in-situ* uniaxial compression tests were conducted at room temperature on the MTS RT/30 Electro-Mechanical Material Testing System controlled by TestWorks 4.0 software, and a high-speed video camera (Canon™ EOS-1D X Mark II) equipped with a telephoto macro lens (Canon™ EF 100–400 mm f/4.5–5.6 L IS II USM Lens with Canon™ 77 mm 500D close-up lens attachment) to observe the deformation behavior of the lattices. Uniaxial compression tests were performed on the microlattices at a prescribed strain rate of 10^−3^ s^−1^. The load-displacement curves were recorded, and by using the mean dimensions in the x-, y-, and z-directions of the lattice structures, the engineering stress and strain were calculated. The compressive strength of the polymer and HEA-coated microlattices were determined by the first applied peak load observed in the stress-strain curve before failure. The fracture morphology of the HEA-coated microlattices were analyzed by using FESEM, in which a thin conductive layer of silver was deposited onto the samples prior to observation to coat the areas with exposed polymer. The Young’s Modulus and elastic recoverability of the microlattices were determined through a series of loading-unloading experiments at strain levels below the fracture strain. All data generated or analyzed during this study are included in this published article.

## Results and Discussion

**Figure 1:**
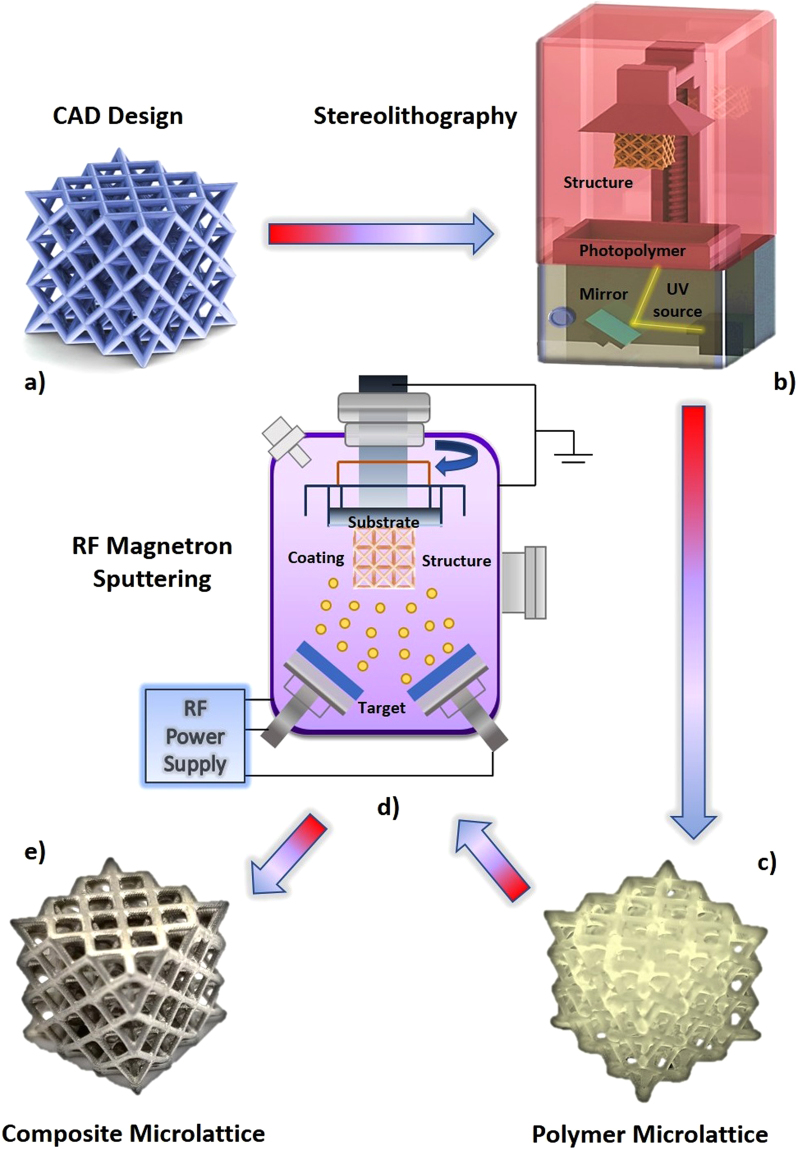
Schematic illustration of the synthesis of HEA-coated microlattices. (**a**) 3D CAD design of the structure used. (**b**) Fabrication process of the 3D polymer template by stereolithography. (**c**) Optical image of the synthesized pure polymer microlattice. (**d**) The HEA thin film coating deposition process onto the polymer structure by RF magnetron sputtering. (**e**) Optical image of the core-shell polymer/HEA microlattice obtained.

Figure 1 shows the schematic illustration of the fabrication process for the 3D core-shell microlattice structure. A CAD model was initially designed using a series of repeated FCC-like unit-cells (Fig. 1a) and subsequently exported into a slicing software prior to 3D printing. A stereolithography (SLA) based printer was used to create the polymer scaffolds (Fig. 1b), allowing features down to the micro-scale to be produced. Quality checking was conducted once the polymer structures were obtained (Fig. 1c) and cleaned sufficiently to enhance the repeatability of the experimental testing results. This involves checking for any observable defects in the printed structure, such as aggregation of cured photopolymer in the pores of the open-cell structure, inconsistent-sized or broken struts, and its overall mass was checked to further confirm the consistency of each sample. As shown in Fig. 1e, the polymer lattices were then coated with a thin layer of HEA by sputter deposition. RF magnetron sputtering was used to conformally coat the lattice in a vacuum environment to finally obtain the core-shell microlattice structure (Fig. 1d).

### Reference HEA Film

**Figure 2:**
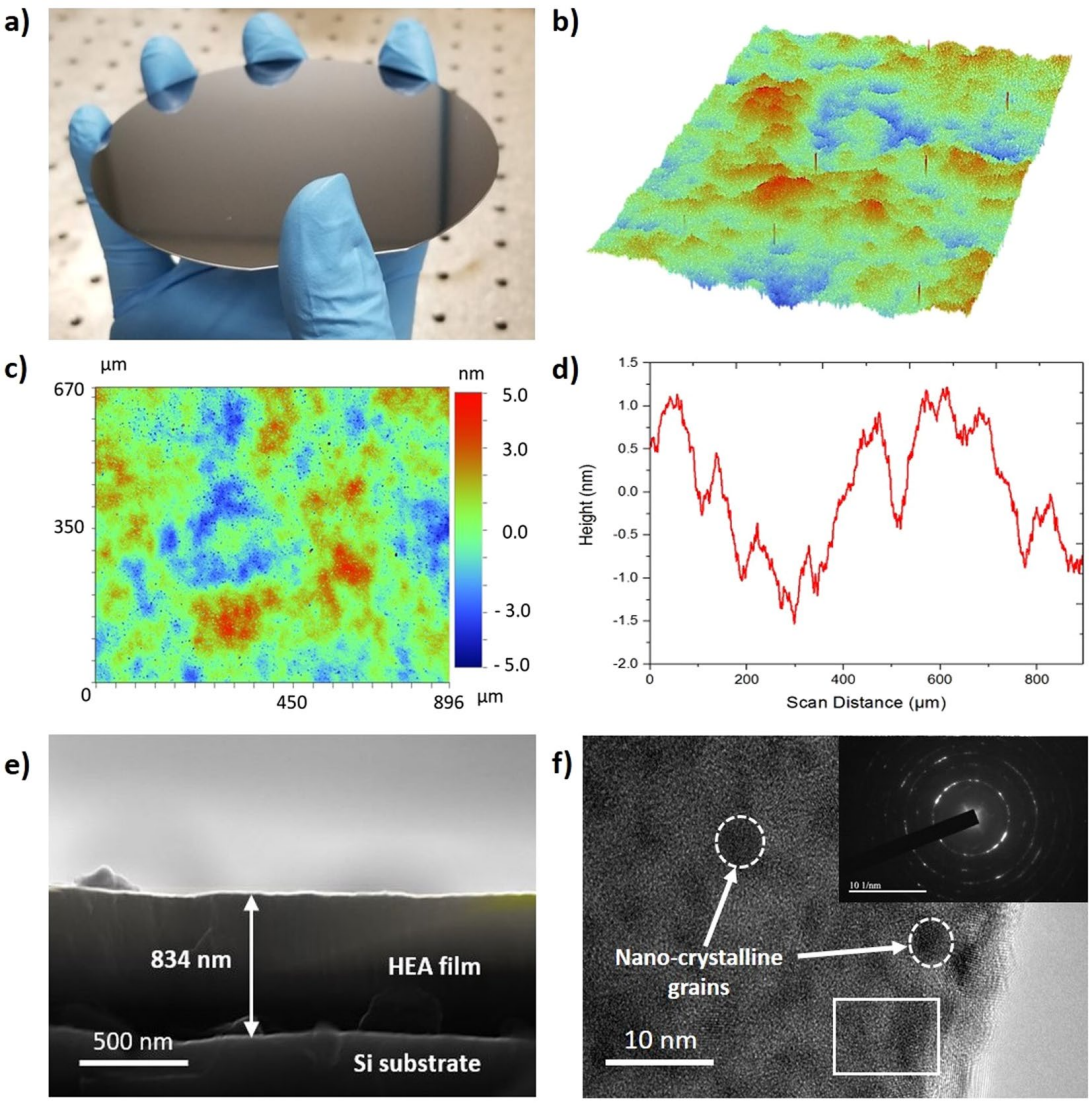
Surface morphology and microstructure of the reference HEA film. (**a**) Optical image of the HEA film deposited onto the silicon substrate. (**b** and **c**) 3D and 2D topographical image of the HEA film surface characterized by WLI. (**d**) Representative 2D surface profile of the HEA film. (**e**) FESEM image of the cross-section view of the reference HEA film. (**f**) Representative TEM image of the HEA film showing the presence of nanocrystalline grains. Inset shows the SAED pattern obtained demonstrating its polycrystalline features, where the white outlined box indicates the selected region.

Figure 2 exhibits the characterization results of the reference HEA film which was deposited onto a standard silicon wafer under the same conditions as the coated microlattices. It can be seen from Fig. 2a that an extremely smooth surface with no obvious signs of aggregation was produced, indicating a uniform deposition of the HEA film onto the silicon substrate macroscopically. A 3D topographical image of the reference film obtained using an analysis software, Vision 4.0, is shown in Fig. 2b. The mean surface roughness (Ra) of the synthesized film, measured at various random positions across the coated substrate using WLI, was approximately 1 nm. This verifies the smooth surface morphology of the as-deposited HEA film A typical 2D image and averaged 2D profile of the HEA film surface is shown in Fig. 2c and d, respectively. The microstructure of the reference HEA film was analyzed by SEM and TEM. The results are shown in Fig. 2e and f.

Figure 2e is the FESEM image of the cross-sectional views of the reference HEA film, respectively. The smooth surface indicates that a homogenous deposition was achieved, and the coating thickness on the silicon substrate is approximately 800 nm. Figure 2f exhibits the representative High Resolution TEM (HRTEM) image of the reference film, which were employed to identify the crystalline features of the HEA film deposited. Small nanocrystalline grains approximately 5 nm in size could be observed, which could be desirable for the mechanical enhancement of the film coating, as the formation of nanocrystalline grains typically result in high strength and hardness for metal and alloys^38,39^. Inset of Fig. 2f shows the corresponding SAED pattern of the HEA film, further confirming its polycrystalline structure. The mechanical characterization of the reference HEA film is shown in Figure S1. Figure S1a exhibits the FESEM image of the smooth top surface of the HEA film, where the insets are the setup and load-displacement curve of a nanoindentation test conducted. Nanoindentation tests (Figure S1b) show that the average Young’s modulus and hardness of the reference HEA film are approximately 180 GPa and 10 GPa, respectively. This is in accordance with previously reported CrMnFeCoNi HEAs^40,41^. The chemical composition of the reference HEA film was analyzed by SEM and EDX (Figure S2). A homogenous elemental composition of Cr, Mn, Fe, Co, and Ni was found, with no observable aggregation of an element in any part of the film (Figure S2a to f). The concentration of each element was also in good agreement with its bulk counterpart (Figure S2g).

### Polymer/HEA-Coated Core-Shell Microlattices

**Figure 3:**
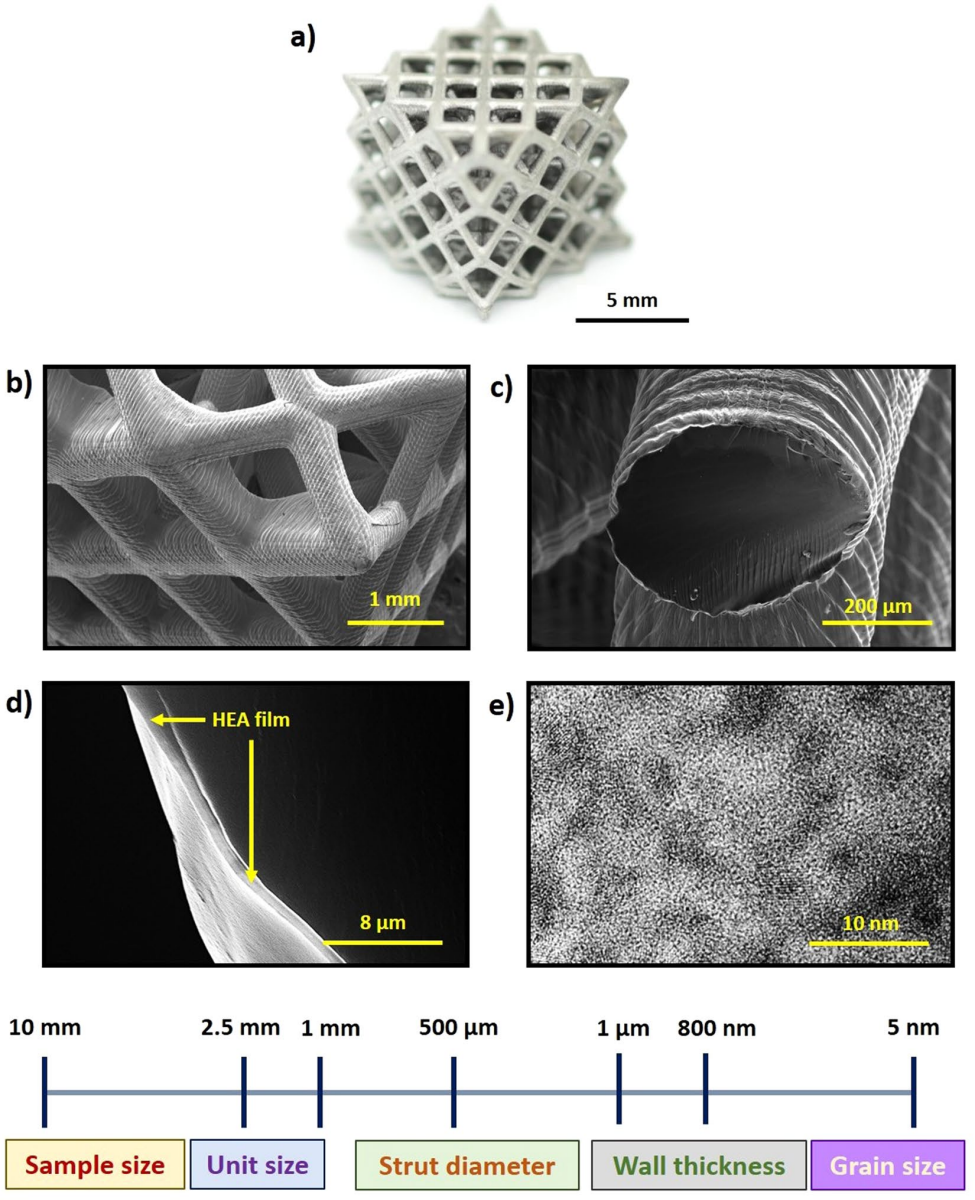
Hierarchical structure of the core-shell microlattice, showing structural features spanning from 5 nm to 75 mm. (**a**) Pure polymer microlattice template. (**b**) HEA-coated microlattice unit. (**c**) A single HEA-coated strut ~450 µm diameter. (**e**) Fractured polymer/HEA microlattice showing the thickness of the HEA coating (~800 nm).

The overall dimensions and morphology of the synthesized microlattices is shown in Fig. 3. Initially, the pristine polymer lattice fabricated by stereolithography, which was designed by a series of repeated open-cell FCC unit cells, made by interconnected struts at its vertices. Each FCC unit cell has a length of approximately 7.50 mm in all the principal axes. The polymer lattices were then conformally coated with a thin layer of HEA film, as exhibited in Fig. 3a. It can be seen from the FESEM image of the HEA-coated microlattice (Fig. 3b) that the coating is uniform, with no obvious voids or defects prior to mechanical testing. The core-shell HEA microlattice structure consist of 12 cylindrical beams or struts that have a diameter of 450 µm (Fig. 3c). Each strut is made up of stacked 2D layers of cured photopolymer coated with a thin layer of HEA, each of which is about 45 µm thick. The post-mortem FESEM image of the coated struts (Fig. 3d) show that the thickness of the HEA film deposited is approximately 800 nm thick, which is in accordance with the reference HEA film used for coating characterization. Figure 3e shows the nanocrystalline grains obtained observed through HRTEM by extraction of the HEA film coating.

**Figure 4:**
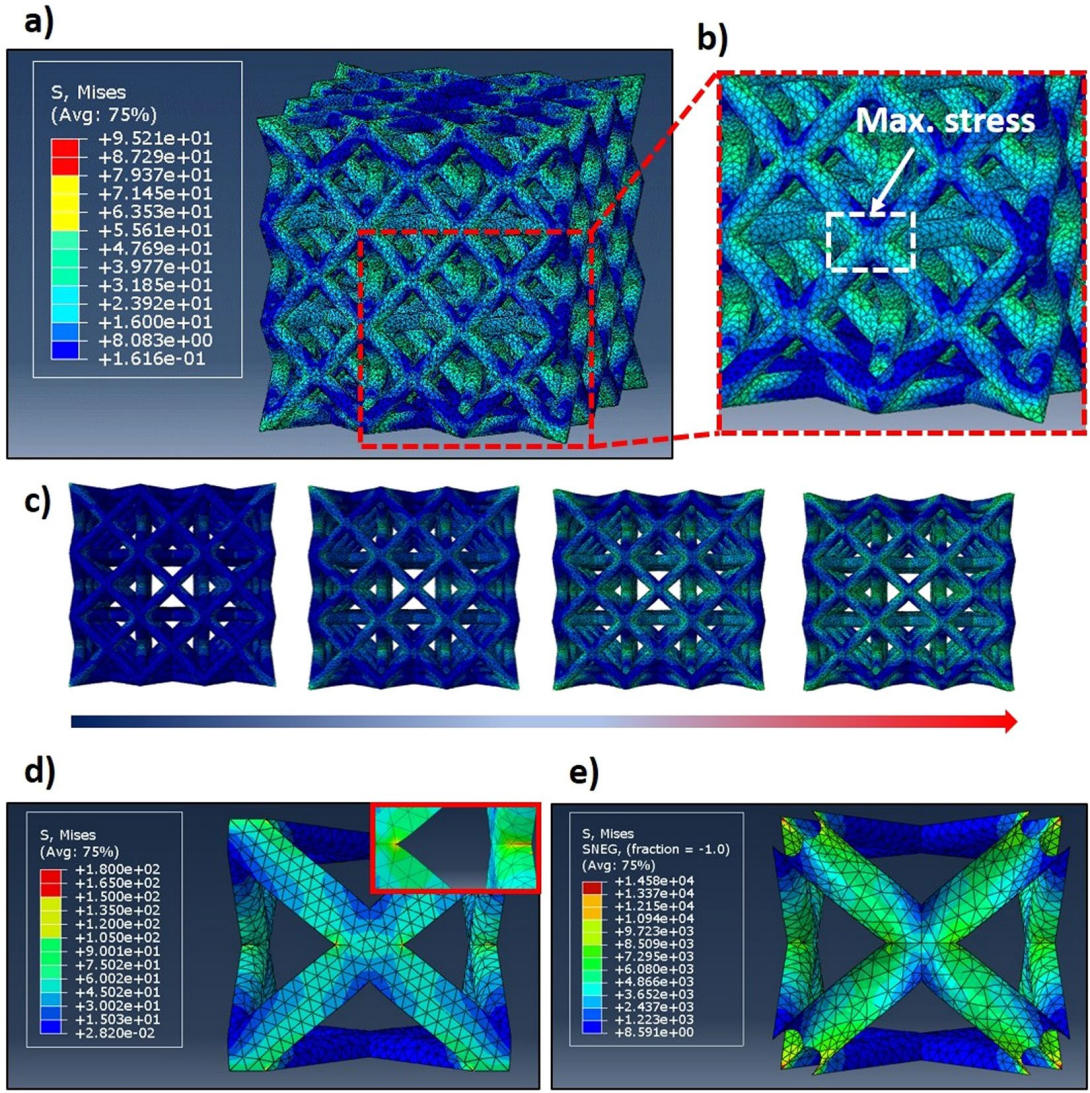
Uniaxial compression tests performed on the polymer and HEA-coated microlattices. (**a**) Layer-by-layer buckling deformation behavior of polymer microlattice. (**b**) Deformation and fracture progression of HEA-coated microlattice during compression. (**c**) Stress-strain curves of the polymer and composite microlattice which were tested under the same environmental and experimental conditions. (**d**) Loading-unloading compression tests performed on the polymer and composite microlattice in the elastic region. (**e** to **h**) Fracture surface morphology of HEA-coated microlattice after compression.

As the core-shell polymer/HEA lattices have been proven to be successfully synthesized, *in situ* uniaxial compression tests were conducted by using a standard mechanical testing equipment. The axial load was applied on the vertical axis, with the microlattice structure placed between the cylindrical metal flat punch. Figure 4a and b shows the optical snapshots obtained during the compression test (at 0%, 5%, 10%, and 50% strain) of the polymer and HEA-coated lattices, respectively. The pristine polymer microlattice exhibits layer-by-layer buckling as it deforms, while the core-shell HEA-coated microlattice deforms through elastic buckling followed by a series of strut fracture in a brittle-like manner at each layer. However, despite the apparent brittle-like fracture mechanism, the strut buckling behavior of the polymer core, as well as the structural configuration prevents catastrophic failure of the HEA-coated microlattice. Figure 4c presents the engineering stress-strain curves for the uniaxial compression test of the microlattices. It can be clearly observed that the core-shell HEA-coated microlattice exhibits a significantly higher compressive strength compared to the pure polymer microlattice. This is attributed to the higher stiffness of the HEA film, causing the film to be the primary external load bearing component. The compressive strength was regarded as the peak stress prior to failure/yielding, and the specific compressive strength achieved for the polymer and composite microlattices were calculated to be 0.003 MPa kg^−1^ m^3^ and 0.018 MPa kg^−1^ m^3^, respectively.

To accurately measure the Young’s modulus and elastic recoverability of the microlattices, a series of loading-unloading uniaxial compression tests were conducted. Based on the results shown in Fig. 4c, it can be predicted that at strain levels up to 4%, the deformation of the polymer and composite microlattice are almost fully elastic. Therefore, the loading-unloading experiments were conducted at 1%, 2%, 3%, and 4% strain, as exhibited in Fig. 4d. It can be clearly seen that the HEA-coated microlattice exhibits a much higher modulus than the pure polymer microlattice, which is attributed to the higher stiffness of the HEA coating. The elastic recoverability of the core-shell microlattice was also found to be better compared to the polymer microlattice at strain levels up to 4%, with no noticeable plastic deformation observed in the case of the HEA-coated microlattice. The slower recovery of the polymer microlattice shown by the hysteresis loop in each of the loading-unloading curves is due to the slight viscoelastic nature of the polymer, whereas such phenomenon is restricted to a considerable extent by the HEA coating for the composite microlattice.

**Figure 5:**
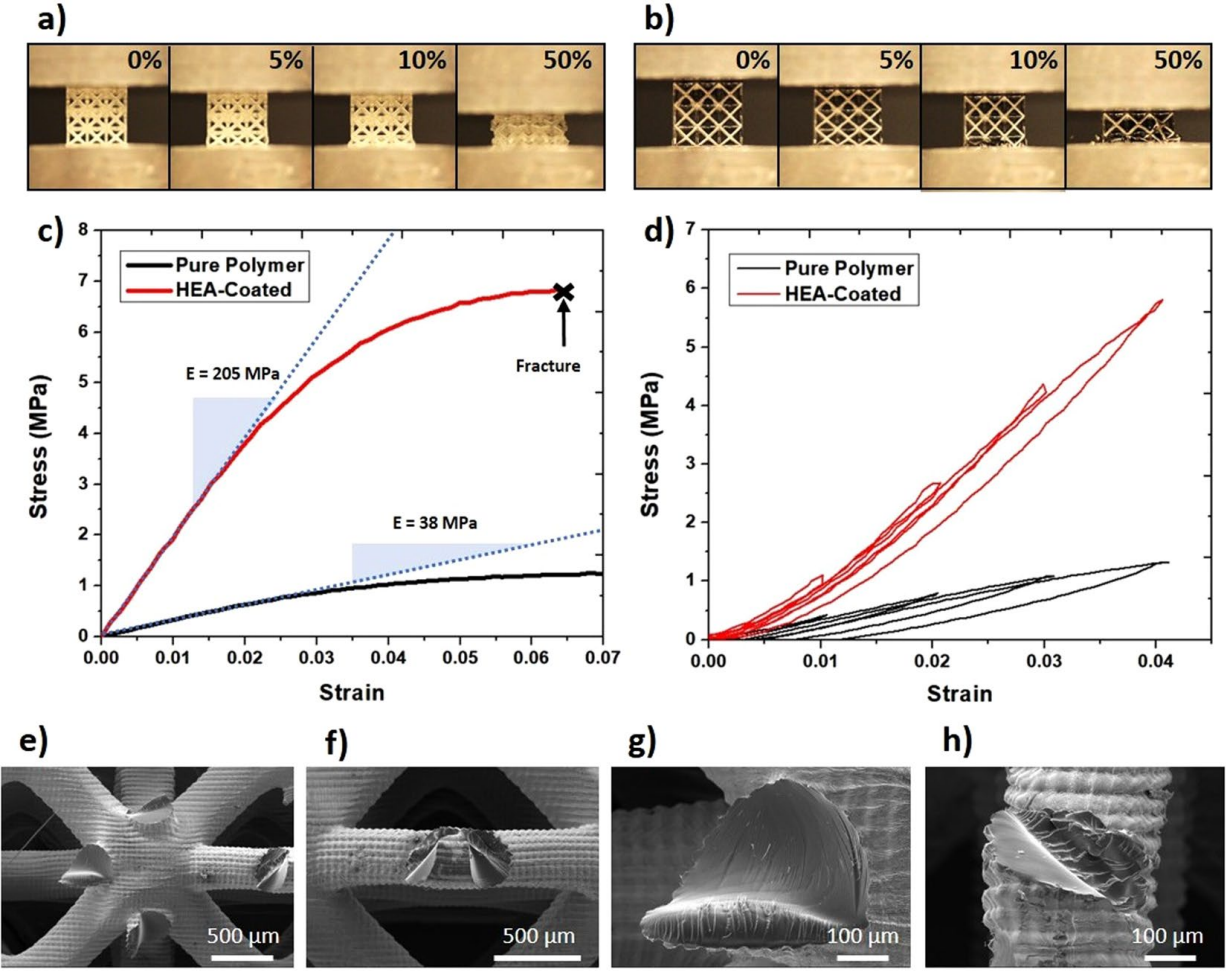
Finite element analysis (FEA) of the polymer and composite microlattices. (**a**) Contour plot of the meshed 3 × 3 × 3 FCC structure using a 10-node quadratic tetrahedral elements after uniaxial compression. (**b**) Enlarged view of the area with highest stress concentration. (**c**) Stress propagation on the FCC microlattice as its compressed. The direction of the arrow indicates the stepwise progression of the displacement from the top surface. (**d** and **e**) Stress distribution on the polymer and HEA coating of the composite microlattice representative unit cell, respectively.

Figure 4e to h shows the FESEM images of the fracture morphology for the HEA-coated microlattice. Figure 4e and f implies that there are two kinds of fracture mechanism, an almost brittle fracture primarily at the vicinity of the node intersections, and a more plastic deformation near the center of the struts. The brittle fracture morphology observed at the proximity of the intersections is akin to that of glass fracture surface when a cylindrical glass beam is subjected to bending. This is potentially caused by an initial crack at the surface of the HEA film due to the maximum stress concentration experienced at the intersection regions, proven by Finite Element Analysis (FEA) of lattice structures (Fig. 5), resulting in the failure of the HEA film^42^. Once the HEA film at one side fails, the crack propagates quickly through the beam as the polymer core could not withstand the stress exerted by the external load. However, a ductile fracture morphology could also be observed on the other side of the beam, as shown in Fig. 4g. This is caused by the reinforcement from the unfractured HEA film, which decelerates the crack propagation, resulting in a transition of the dominating fracture mechanism to viscoplastic yielding. The elastic beam buckling, brittle fracture, and viscoplastic yielding deformation mechanisms of the microlattice have all been observed previously. Furthermore, the brittle nature of the HEA film coating is due to the ductile-to-brittle size effect observed in previously reported microlattices^8,13,43^. The second type of fracture surface (Fig. 4h), in which a rugged morphology can be seen along with film removal, is more unconventional and rarely seen, observable only in a small quantity of the samples tested. This is probably caused by small defects or cracks in the film surface, which act as stress concentrators. Thus, when the external load was applied, the cracks in the film will propagate along the crack tip, causing the HEA film to be “peeled-off” from the surface of the polymer core. Subsequently, polymer beam will undergo viscoplastic yielding due to the shearing force.

Throughout the years, thin film coatings, whether amorphous or crystalline, have been extensively studied and proven to be able to significantly enhance the mechanical performance of a wide variety of lower density materials. In our case, the HEA film coating deposited onto the polymer scaffold by sputter deposition could increase the compressive strength of the polymer by approximately 6 times. The mechanically enhanced strength of the polymer/HEA core-shell microlattice, achieved through the combination of stretching-dominated architecture and HEA coating, is higher than other composite microlattices, such as polymer/NiB (0.016 MPa kg^−1^ m^3^)^13^, polymer/NiP (0.007 MPa kg^−1^ m^3^)^44^, polymer/SiO_2_ (0.003 MPa kg^−1^ m^3^)^45^, and polymer/Si_3_N_4_ (0.012 MPa kg^−1^ m^3^)^15^. This demonstrates the potential of HEA films as coatings for the development of high-strength and lightweight hybrid microlattices with tunable mechanical properties for structural applications. Additionally, HEAs have been proven to be able to display unique properties for specialized applications. For instance, the CrMnFeCoNi HEA used in this work has been previously reported to have exhibited enhanced strength, ductility, and toughness at cryogenic temperatures, whereas most materials transition from ductile to brittle deformation behavior^27,28,46^.

## Conclusion

This work presents the successful fabrication and mechanical characterization of the first core-shell HEA-coated microlattice metamaterial with critical feature sizes spanning from nano- to micro- to bulk scales (5 nm to 75 mm). The hybrid microlattices were synthesized by employing RF magnetron sputtering technology to deposit a thin layer of CrMnFeCoNi HEA film onto the micro-architected polymer scaffold obtained by stereolithography. *In-situ* uniaxial compression experiments on the core-shell microlattices revealed its characteristic mechanical deformation and fracture behavior. The high specific compressive strength of the coated microlattice achieved (~6 times higher than pure polymer) with density significantly below 1000 kg m^−3^ highlights the advantages of combining HEA with low density architected polymer lattices to develop alterable mechanical metamaterials by chemical composition and structural variations. This design concept, along with the potential high scalability of the light-based 3D printing process in future manufacturing larger-scale products with micro-scale features, offers the potential to be applied in the field of structural engineering and the development of robust flexible electronics or biomedical instruments.

## Electronic supplementary material

Supplementary Information
