# Supplementary material for: Mechanical Enhancement of Core-Shell Microlattices through High-Entropy Alloy Coating
Source: Sci Rep. 2018 Apr 3;8:5442. doi: 10.1038/s41598-018-23857-7 (PMC5882655; doi:10.1038/s41598-018-23857-7)
Supplement: Supplementary file 1 — Supplementary Information [file 41598_2018_23857_MOESM1_ESM.pdf]

Supplementary Information

Mechanical Enhancement of Core-Shell Microlattices through High-Entropy Alloy (HEA) Coating

James Utama Surjadi[1ψ], Libo Gao[1,2ψ], Ke Cao[1], Rong Fan[1], Yang Lu[1,2*]

[1]Department of Mechanical and Biomedical Engineering, City University of Hong Kong, Kowloon, Hong Kong

[2]Shenzhen Research Institute, City University of Hong Kong, Shenzhen 518057, China

ψ These authors contributed equally to this work.

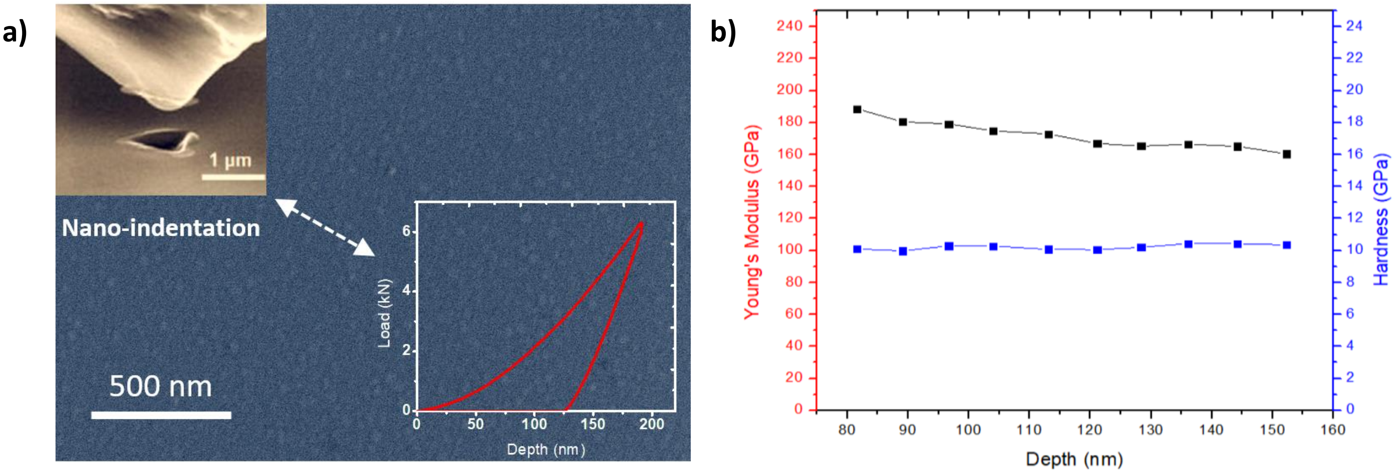

Figure S1. Mechanical characterization of the reference HEA film. a) FESEM image of the top view of the reference HEA film with an illustration of the nanoindentation test setup and an example load-displacement curve. b) Nanoindentation test results showing the Young's modulus and hardness of the reference HEA film at different depths.

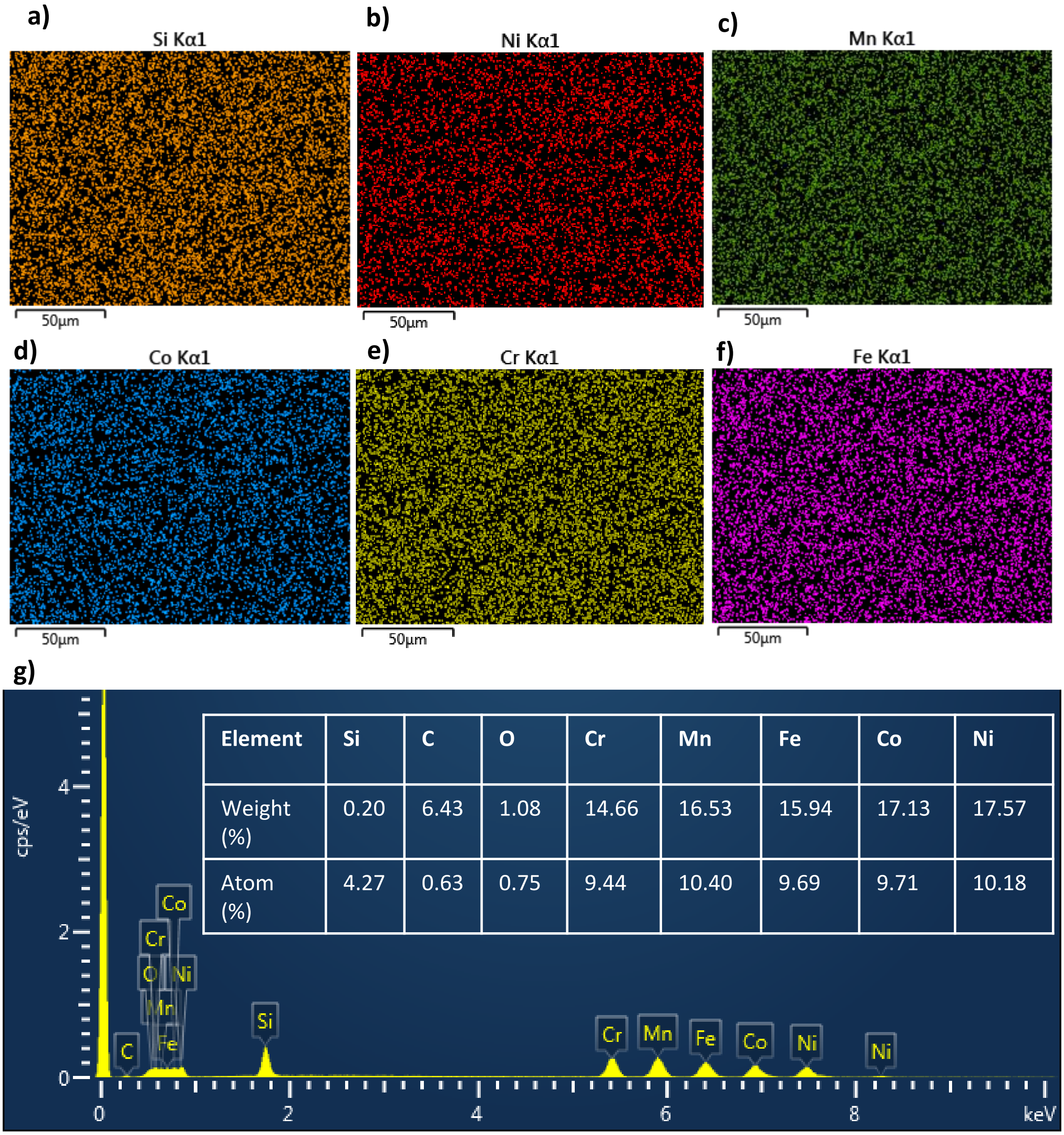

Element	Si	C	O	Cr	Mn	Fe	Co	Ni
Weight (%)	0.20	6.43	1.08	14.66	16.53	15.94	17.13	17.57
Atom (%)	4.27	0.63	0.75	9.44	10.40	9.69	9.71	10.18

Figure S2. SEM/EDX mapping and elemental composition of the reference HEA film.

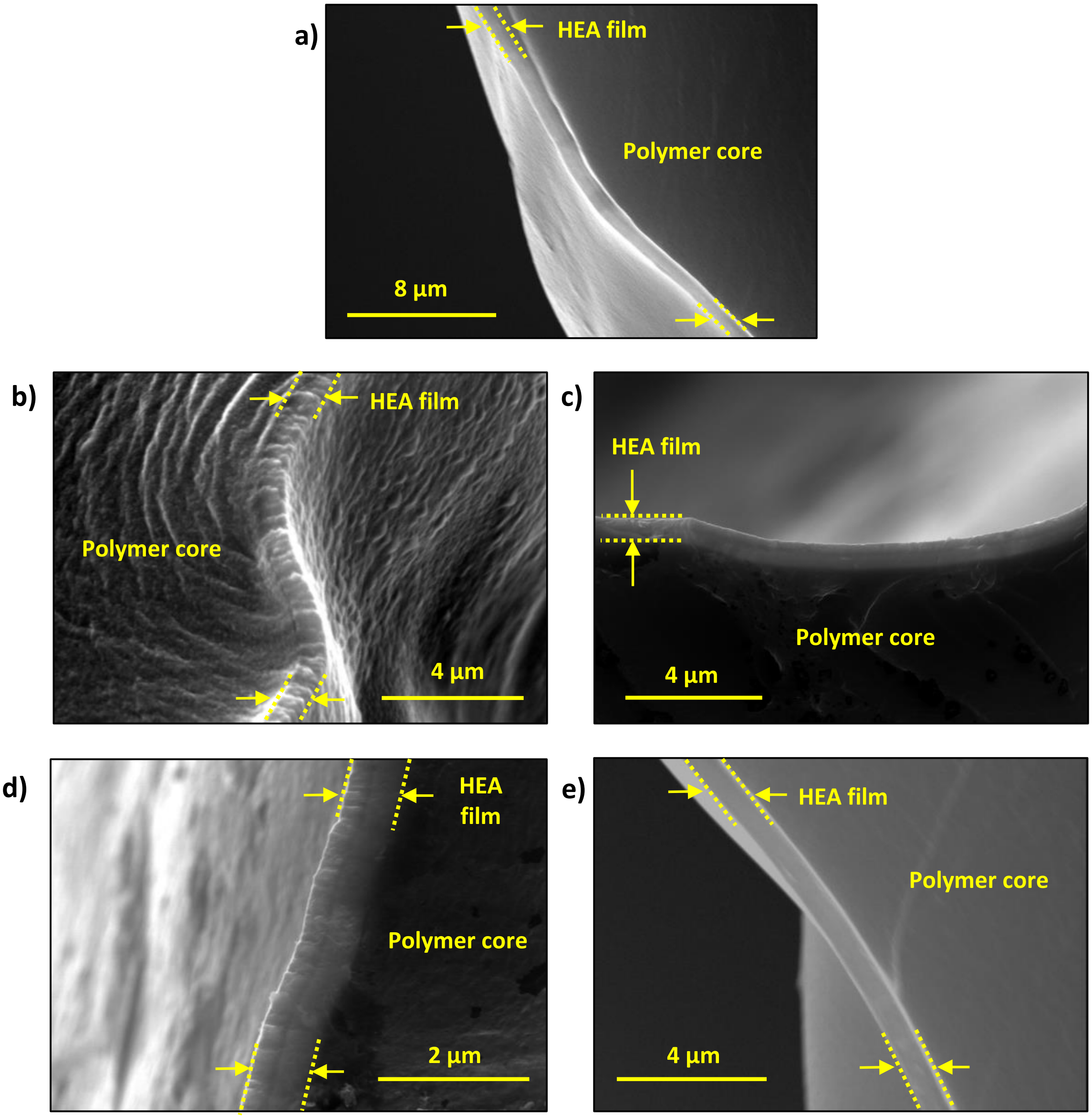

Figure S3. SEM images showing the thicknesses of the HEA film deposited on the polymer microlattice at various places within the structure.

Sample	Structure	Coating thickness, t (nm)	Strut diameter, d (µm)	t/d	Peak strength (MPa)	Specific strength (MPa m³/kg)	Young's modulus (MPa)	Compressive strain (%)
**Polymer/ NiB** [1]	Cubic-truss	~ 30	~ 1	~ 0.0300	~ 1.94	~ 0.016	~ 116	3
**Polymer/ NiP** [2]	Diamond-structured	~ 1600	~ 300	~ 0.0053	~ 1.25	~ 0.007	~ 30	5
**Polymer/ SiO_2** [3]	Diamond-structured	~ 2360	~ 800	~ 0.0030	~ 0.40	~ 0.003	~ 8	9
**Polymer/ Si_3N_4** [4]	Diamond-structured	~ 400	~ 300	~ 0.0013	~ 1.90	~ 0.012	~ 40	10
**Polymer/ HEA (Our work)**	FCC-structured	~ 800	~ 450	~ 0.0018	~ 6.90	~ 0.018	~ 205	7

Table S1. Comparison of proposed HEA-coated microlattice with previously reported similar works.

References:

1. Mieszala, M. *et al.* Micromechanics of Amorphous Metal/Polymer Hybrid Structures with 3D Cellular Architectures: Size Effects, Buckling Behavior, and Energy Absorption Capability. *Small* **13,** 1–13 (2017).

2. Fan, Q. *et al.* Fabrication of diamond-structured composite materials with Ni-P-diamond particles by electroless plating. *Mater. Lett.* **215,** 242–245 (2018).

3. Wei, F., Zhou, Y., Yang, Q., Guo, L. & Jiang, L. Silica film deposited on diamond-structured polymer microlattices by dip coating. *RSC Adv.* **7,** 54668–54673 (2017).

4. Zhou, Y. F., Yao, C. Z., Yang, Q. L., Guo, L. & Jiang, L. Mechanical Properties of Diamond-Structured Polymer Microlattices Coated with the Silicon Nitride Film. *Adv. Eng. Mater.* **18,** 236–240 (2016).
